# Algorithmically Deduced *FREM2* Molecular Pathway Is a Potent Grade and Survival Biomarker of Human Gliomas

**DOI:** 10.3390/cancers13164117

**Published:** 2021-08-16

**Authors:** Marianna Zolotovskaia, Victor Tkachev, Maxim Sorokin, Andrew Garazha, Ella Kim, Sven Rainer Kantelhardt, Sven-Ernö Bikar, Alja Zottel, Neja Šamec, Denis Kuzmin, Bettina Sprang, Alexey Moisseev, Alf Giese, Victor Efimov, Ivana Jovčevska, Anton Buzdin

**Affiliations:** 1 Omicsway Corp., Walnut, CA 91789, USA; sorokin@oncobox.com (M.S.); garazha@oncobox.com (A.G.); moiseev_a_a@staff.sechenov.ru (A.M.); 2 Moscow Institute of Physics and Technology, Dolgoprudny 141701, Russia; tkachev@oncobox.com (V.T.); kuzmin.dv@mipt.ru (D.K.); ef.viktor12@gmail.com (V.E.); buzdin.aa@mipt.ru (A.B.); 3 Department of Oncology, Hematology and Radiotherapy, Pirogov Russian National Research Medical University, Moscow 117997, Russia; 4 Laboratory of Clinical Genomic Bioinformatics, I.M. Sechenov First Moscow State Medical University, Moscow 119991, Russia; 5 Clinic for Neurosurgery, Laboratory of Experimental Neurooncology, Johannes Gutenberg University Medical Centre, Langenbeckstrasse 1, 55124 Mainz, Germany; ella.kim@unimedizin-mainz.de (E.K.); sven.kantelhardt@unimedizin-mainz.de (S.R.K.); bettina.sprang@unimedizin-mainz.de (B.S.); 6 StarSEQ GmbH, Joh.-Joachim-Becher-Weg 30a, 55128 Mainz, Germany; Bikar@starseq.com; 7 Medical Center for Molecular Biology, Institute of Biochemistry and Molecular Genetics, Faculty of Medicine, University of Ljubljana, Vrazov Trg 2, 1000 Ljubljana, Slovenia; alja.zottel@mf.uni-lj.si (A.Z.); neja.samec@mf.uni-lj.si (N.Š.); ivana.jovcevska@mf.uni-lj.si (I.J.); 8 Orthocentrum Hamburg, Hansastrasse 1, 20149 Hamburg, Germany; prof.giese@oc-h.de; 9 Shemyakin-Ovchinnikov Institute of Bioorganic Chemistry, Moscow 117997, Russia; 10 European Organization for Research and Treatment of Cancer (EORTC), Biostatistics and Bioinformatics Subgroup, 1200 Brussels, Belgium

**Keywords:** *FREM2*, glioma, glioblastoma, survival prognosis, algorithmically deduced molecular pathway, transcriptomics

## Abstract

**Simple Summary:**

Gliomas are the most common malignant brain tumors with high mortality rates. Recently the role of the *FREM2* gene has been shown in glioblastoma progression. Here we reconstructed the *FREM2* molecular pathway. We assessed the biomarker capacity of *FREM2* expression and its pathway as the overall survival (OS) and progression-free survival (PFS) biomarkers. We used 566 glioblastomas (GBM) and 1097 low-grade gliomas (LGG) to test these biomarkers. *FREM2* molecular pathway was a better biomarker than *FREM2* gene expression. It could robustly discriminate between GBM and LGG. High *FREM2* pathway activation level was associated with poor overall survival (OS) in LGG, and low progression-free survival in LGG and GBM. *FREM2* pathway activation level was also a poor prognosis biomarker for OS and PFS in LGG with *IDH* mutation, for PFS in LGG with wild type *IDH* and mutant *IDH* with 1p/19q codeletion, in GBM with unmethylated *MGMT*, and in GBM with wild type *IDH*.

**Abstract:**

Gliomas are the most common malignant brain tumors with high mortality rates. Recently we showed that the *FREM2* gene has a role in glioblastoma progression. Here we reconstructed the *FREM2* molecular pathway using the human interactome model. We assessed the biomarker capacity of *FREM2* expression and its pathway as the overall survival (OS) and progression-free survival (PFS) biomarkers. To this end, we used three literature and one experimental RNA sequencing datasets collectively covering 566 glioblastomas (GBM) and 1097 low-grade gliomas (LGG). The activation level of deduced *FREM2* pathway showed strong biomarker characteristics and significantly outperformed the *FREM2* expression level itself. For all relevant datasets, it could robustly discriminate GBM and LGG (*p* < 1.63 × 10^−13^, AUC > 0.74). High *FREM2* pathway activation level was associated with poor OS in LGG (*p* < 0.001), and low PFS in LGG (*p* < 0.001) and GBM (*p* < 0.05). *FREM2* pathway activation level was poor prognosis biomarker for OS (*p* < 0.05) and PFS (*p* < 0.05) in LGG with *IDH* mutation, for PFS in LGG with wild type *IDH* (*p* < 0.001) and mutant *IDH* with 1p/19q codeletion(*p* < 0.05), in GBM with unmethylated *MGMT* (*p* < 0.05), and in GBM with wild type *IDH* (*p* < 0.05). Thus, we conclude that the activation level of the *FREM2* pathway is a potent new-generation diagnostic and prognostic biomarker for multiple molecular subtypes of GBM and LGG.

## 1. Introduction

Gliomas account for ~30% of all CNS tumors and 80% of all malignant brain tumors [1,2]. In the current WHO classification, there are four grades of glioma (I–IV) that reflect pathological evaluation and molecular characteristics of a tumor [3]. The grade has typical survival characteristics and impacts on the treatment approach. The most advanced grade (IV) includes malignant glioblastoma multiforme (GBM) tumors. GBM is the most common malignant brain tumor in adults with an age-adjusted annual incidence rate of 0.6–3.7 per 100,000 individuals [4,5]. The median overall survival (OS) of GBM patients is only 12 months [6], and GBM remains a treatable but incurable disease with inevitable lethal outcomes [7]. GBM has a heterogeneous origin, aggressive nature, quick progression, and occurs in vitally important tumor sites which complicates surgery and radiation therapy applications [5,8].

Other types of gliomas (grade 1–3) are typically called low-grade gliomas (LGG), while some authors refer to LGG only grade 1–2 gliomas and other authors refer to LGG diffuse low-grade and intermediate-grade gliomas (grade 2–3) [9]. In this study, we took grade 2–3 gliomas for the “LGG” group. Some LGG cases can rapidly transform into GBM within months, whereas the others can remain stable for years. Correspondingly, mean OS varies for different LGG subgroups from 1 to ~15 years, and a fraction of LGGs is highly sensitive to the therapy [9].

Furthermore, molecular markers can serve as predictors for the survival of glioma patients. Robust biomarkers such as *IDH* mutation and *MGMT* promoter methylation are associated with better survival in gliomas [9,10,11]. A meta-analysis of 55 studies involving 9487 patients with gliomas showed that patients with the *IDH* mutation had better overall survival (Hazard ratio 0.39, 95% CI: 0.34–0.45; *p* < 0.001) and progression-free survival (Hazard ratio 0.42, 95% CI: 0.35–0.51; *p* < 0.001) [12]. Median OS for wt*IDH* LGGs (1.7 years) is between OS for wt*IDH* GBMs (1.1 years) and mutated *IDH* GBMs (2.1 years) [9]. *MGMT* promoter methylation was associated with longer PFS and OS in GBM patients without therapy, and with better OS in GBM patients treated by DNA-alkylating agents such as temozolomide [13,14]. In turn, methylated *MGMT* promoter is also a favorable predictor of PFS in LGG treated with neoadjuvant temozolomide [11].

Recently, using agnostic proteomic screening we found that *FREM2* (FRAS1 Related Extracellular Matrix 2) gene product was statistically significantly associated with GBM [15,16]. *FREM2* encodes an integral membrane protein with multiple chondroitin sulfate proteoglycan element (CSPG) repeats and Calx-beta domains that mark sodium-calcium exchanger activity, which is used to expel calcium from cells. The expression of *FREM2* is higher in GBM cell lines than in normal astrocytes [15]. Also, *FREM2* gene and protein expression levels are higher in GBM stem cells compared to conventional GBM cell lines [15]. This trend was also confirmed on human tumor tissue samples. Increased *FREM2* expression was found in LGGs compared to healthy brain tissues, and in GBMs compared to LGGs [16].

In this study, we analyzed *FREM2* gene expression and its linkage with survival using the major publicly available datasets of LGG and GBM RNA sequencing profiles. We also reconstructed the *FREM2* molecular pathway using the human interactome model and found that it had a significantly better performance as the OS and PFS biomarker of gliomas. To this end, we used three literature and one experimental RNA sequencing datasets collectively covering 566 glioblastomas (GBM) and 1097 low-grade gliomas (LGG). The activation level of deduced *FREM2* pathway showed strong biomarker characteristics and significantly outperformed the *FREM2* expression level itself. For all relevant datasets, it could discriminate GBM and LGG (*p* < 1.63*10^−13^, AUC > 0.74). High *FREM2* pathway activation level was associated with poor prognosis in LGG and GBM and several LGG or GBM subtypes. Thus, we conclude that the activation level of the *FREM2* pathway is a potent new-generation diagnostic and prognostic biomarker for multiple molecular subtypes of GBM and LGG.

## 2. Materials and Methods

### 2.1. The Cancer Genome Atlas (TCGA) Dataset

Overall and progression-free survival data were extracted from the clinical description on the GDC Data Portal for 591 GBM and 510 LGG samples [17]. RNA sequencing (RNAseq) data (HTseq counts) were downloaded from the GDC Data Portal [17]. Only primary tumor samples were selected (153 and 505 samples, respectively). *MGMT* methylation statuses were obtained for GBM samples from the report [18] and LGG samples from the report [19]. *IDH* mutation statuses were extracted for GBM and LGG samples from SNV data (vcf files) from the GDC Data Portal. Molecular subtype classifications were obtained from Ceccarelli M. et al. [19] and the GlioVis portal (http://gliovis.bioinfo.cnio.es/, acceded on 7 August 2021).

### 2.2. The Chinese Glioma Genome Atlas Dataset CGGA_325

Overall survival data, *IDH* mutation status, *MGMT* methylation status, and patient age information were extracted from the clinical description for 137 GBM and 172 LGG samples from the CGGA database; dataset id: mRNAseq_325 [20,21]. RNAseq data (RSEM counts) were downloaded from the CGGA database for the corresponding biosamples. Molecular subtype classification was obtained from the GlioVis portal (http://gliovis.bioinfo.cnio.es/, accessed on 7 August 2021).

### 2.3. The Chinese Glioma Genome Atlas Dataset CGGA_693

Overall survival data, *IDH* mutation status, *MGMT* methylation status, and patient age information were extracted from the clinical description for 237 GBM and 420 LGG samples included in CGGA database; dataset id: mRNAseq_693) [22,23]. RNAseq data were downloaded from the CGGA database for the corresponding samples. Molecular subtype classification was obtained from the GlioVis portal (http://gliovis.bioinfo.cnio.es/, accessed on 7 August 2021).

### 2.4. The Experimental Dataset

#### 2.4.1. Biosamples

Thirty-nine tumor samples were collected from 16 patients with primary GBM who were operated on at the Johannes Gutenberg University Medical Center Mainz (UMM). For some experimental patients (12/16), two or more tumor samples were included in the analysis that were obtained surgically from different regions of the same tumor. Written informed consents for using excess tumor tissue for research purposes were obtained from all the patients. Tumor samples were coded and processed for RNAseq anonymously and in accordance with the approval by the UMM Institutional Review Board and ethics committee approval No. 837.178.17 (11012) granted to the UMM Clinic for Neurosurgery by the Rhineland Palatinate Chamber of Physicians (Landesäzrtekammer Rheinland-Pfalz, https://www.laek-rlp.de/ausschuesse-kommissionen/ethikkommission/, accessed on 17 April 2021). Clinical data were obtained for every patient including diagnosis, *IDH* mutation status, *MGMT* promoter methylation status, type of therapy, and time to progression (Supplementary File 1).

#### 2.4.2. RNA Sequencing

Preparation of RNAseq libraries was performed as described previously [24]. Frozen GBM samples were homogenized. RNA was extracted using the Precellys Tissue RNA Kit Safety-Line (Peqlab) according to the manufacturer’s protocol. RNA integrity number (RIN) was measured using Agilent 2100 Bioanalyzer with Agilent RNA 6000 pico and nano assay. RNA concentration was measured using Qubit 2 and Qubit 4 fluorometers (Invitrogen, Baden-Württemberg, Germany) with RNA BR and HS assay kits. Samples with an RNA integrity number (RIN) less than seven were excluded from subsequent library preparation. For generating libraries, we used the TruSeq Stranded Total RNA Library Prep Kit ((Illumina, Berlin, Germany) and the NEBNext Ultra II Directional RNA Library Prep Kit (New England BioLabs, Frankfurt am Main, Germany) according to the manufacturer protocols. Different indexing adaptors were used for multiplexing of samples in one sequencing run. Library concentrations were measured using Qubit dsDNA high-sensitivity (HS) kit and QIAxcel capillary electrophoresis system with QIAxcel ScreenGel software (Qiagen, Hilden, Germany). Paired-end RNA sequencing was carried out at StarSEQ laboratory using Illumina NextSeq 500 engine, 150 bp read length, for approximately 25–30 million reads per sample. A data quality check was done using Illumina SAV and FastQC software. De-multiplexing was performed using Illumina bcl2fastq2 software. RNAseq FASTQ files were processed with STAR aligner [25] and annotated with HGNC identifiers. The 23582-gene expression profile was obtained for every sample under analysis, statistics of reads mapping is given in Supplementary File 2. Gene expression profiles (raw counts) were deposited in the Gene Expression Omnibus database (GEO) under accession number GSE139533.

### 2.5. Source Molecular Pathways

The gene structures and molecular architectures of 1180 intracellular pathways were extracted from the publicly available databases Reactome [26], NCI Pathway Interaction Database [27], Biocarta [28], and Qiagen [29] as described in [30].

### 2.6. Pathway Activation Level Calculation

*Pathway activation level (PAL)* is an aggregated quantitative and qualitative characteristic of changes in expression levels for genes participating in a certain molecular pathway [30,31,32]. PALs were calculated as follows:

$$PAL_{p}=\sum_{n}ARR_{np}*\mathrm{lg}\left(CNR_{n}\right)/\sum_{n}|ARR_{np}|**100,$$

where *PAL_p_* is *PAL* for pathway p, *CNR_n_* is the *case-to-normal ratio*, the ratio of gene *n* expression level in a sample understudy to an average level in the control group; *ARR* (*activator/repressor role*) is a Boolean value that depends on the function of this gene product in pathway *p*. ARR values were defined as follows: −1 when the product of *n* inhibits *p*; 1 when *n* activates *p*; 0 when *n* has an ambiguous or unclear role in the pathway; 0.5 or −0.5, when *n* is rather *p* activator or inhibitor, respectively. We used an averaged-by-gene-expression tumor sample as the control. PAL values calculated for all the samples investigated are available in Supplementary File 3.

### 2.7. Statistical Analysis

We used principal component analysis to assess the compatibility of glioma gene expression data from a different dataset. Also, quality measuring of hierarchical clustering was applied to CGGA datasets for the same goal. This was performed by the *Watermelon multisection* method [33] that returns WM metric which positively reflects the quality of clustering of samples into pre-defined groups.

ROC AUC value and *t*-test were used as the measure of biomarker quality in comparison of low-grade glioma with glioblastomas.

Overall and progression-free survival was assessed by Kaplan-Meyer analysis. The statistical significance of survival differences was measured with a log-rank test *p*-value. Hazard ratios were calculated in the univariate and multivariate Cox model to assess survival differences in comparative groups.

## 3. Results

### 3.1. Glioma Expression Datasets

We used three major publicly available glioma datasets with clinically annotated RNA sequencing profiles (from TCGA and CGGA databases [17,20,21,22,23]), and one experimental dataset [24]. These datasets were classified into subsets including glioblastoma (GBM), or low-grade glioma (LGG) samples. Only samples with survival data were included in the analysis, thus totaling 566 GBM and 1097 LGG RNAseq profiles.

Specifically, the TCGA dataset included 153 GBM (mean age 60 y.o., 99 male and 54 female patients), and 505 LGG samples (mean age 43 y.o., 279 male and 226 female patients), and overall survival (OS), progression-free survival (PFS) [17], *MGMT* promoter methylation (only for GBM samples) [18], and *IDH* mutation statuses were extracted [17].

CGGA database contained two large RNAseq glioma datasets with internal IDs *mRNAseq_325* and *mRNAseq_693* [20,21,22,23], which were referred to here as CGGA_325 and CGGA_693, respectively. OS, *IDH* mutation, and *MGMT* methylation status information were available for both datasets. CGGA_693 contained RNAseq profiles for 237 GBMs (mean age 49 y.o., 139 male and 98 female patients) and for 420 LGGs (mean age 40 y.o., 235 male and 185 female patients).

CGGA_325 included profiles for 137 GBMs (mean age 47 y.o., 87 male and 50 female patients) and 172 LGGs (mean age 40 y.o., 106 male and 66 female patients).

The experimental dataset included 39 tumor samples, which were collected from 16 patients with primary GBM (mean patient age at the date of diagnosis 60 y.o, 9 male and 7 female patients). Some patients (12/16) have several tumor samples which were obtained from different regions of the same tumor. RNAseq profiles were deposited in Gene Expression Omnibus (GEO) database under accession number GSE139533. PFS, *IDH* mutation, and *MGMT* promoter methylation information was collected for each patient, but no OS information was available.

### 3.2. Compatibility of Glioma Gene Expression Data

**Figure 1:**
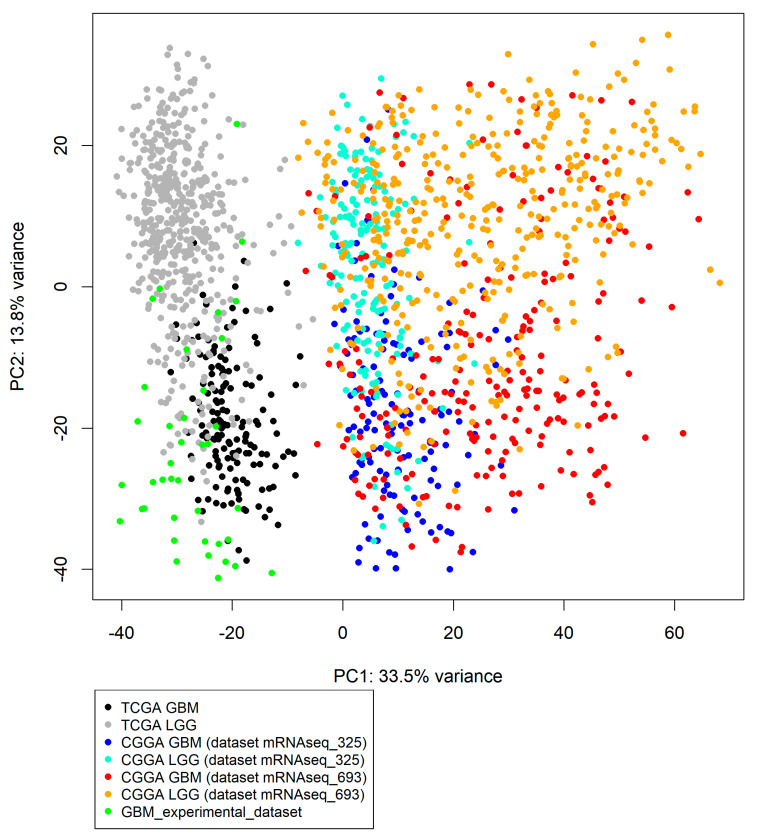
Principle component analysis (PCA) plot built for primary gene expression data of three literature datasets for LGG, tree literature datasets for GBM, and one experimental dataset for GBM samples. PCA plot was built using prcomp function from stats R package [35].

The expression data from the datasets under investigation were all processed using the Illumina platform but were obtained with different library preparation kits and protocols. Thus, all datasets had a different number of sequenced genes (36304, 24326, 23987, 23582 for TCGA, CGGA_325, CGGA_693, and experimental datasets, respectively). We normalized raw counts by DESeq2 software [34] and inspected the data using principal component analysis (PCA) that showed significantly different patterns for all literature datasets when separately comparing LGG and GBM samples, respectively (Figure 1).

In addition, hierarchical clustering of samples from two batches of the CGGA project (CGGA_325 and CGGA_693) showed that samples were clustering by batch ID rather by glioma type (LGG or GBM). This was also quantitatively measured by the *Watermelon multisection* method [33] that returns WM metric which positively reflects the quality of clustering of samples into pre-defined groups, with WM metrics 0.839 and 0.159 for batch- and glioma type clustering, respectively (data not shown).

Thus, we didn’t combine CGGA_325 and CGGA_693 expression data and used them as the two independent literature datasets.

### 3.3. Human Interactome Model

**Figure 2:**
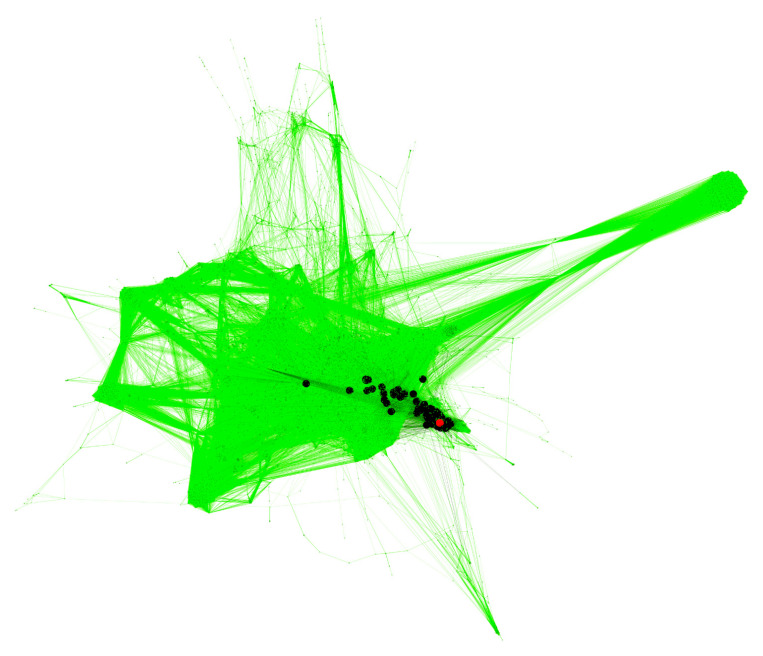
Human interactome model. Genes (gene products) are nodes and protein-protein interactions are edges of the graph. Black dots represent gene products involved in the *FREM2* pathway corresponding to PAL2, and the red dot denotes the *FREM2* gene product.

Using a collection of published molecular pathways as the knowledge base of molecular interactions, we built a human interactome model—the graph, where the nodes are genes/gene products, and the edges are known pairwise connections between the elements of molecular pathways. Visualization of the model was performed using Gephi software, ForceAtlas2 algorithm [36] (Figure 2).

Molecular architectures of 1180 different pathways were used. Gene composition and nodes interactions of the pathways were extracted and cataloged. We combined all pathway graphs based on the coinciding genes/gene products. The obtained interactome graph consists of 7152 nodes (genes/gene products) with 298,824 molecular interactions. The following types of interactions were considered: “activation”, “compound”, “inhibition”, “phosphorylation”, “dissociation”, “repression”, “dephosphorylation”, “binding/association”, “ubiquitination”. The graph has a low density (0.01) with an average vertex degree of 42. All genes included form a connected network, i.e., there is an at least undirected path between every pair of genes/gene products involved.

### 3.4. Reconstruction of FREM2 Molecular Pathway

**Figure 3:**
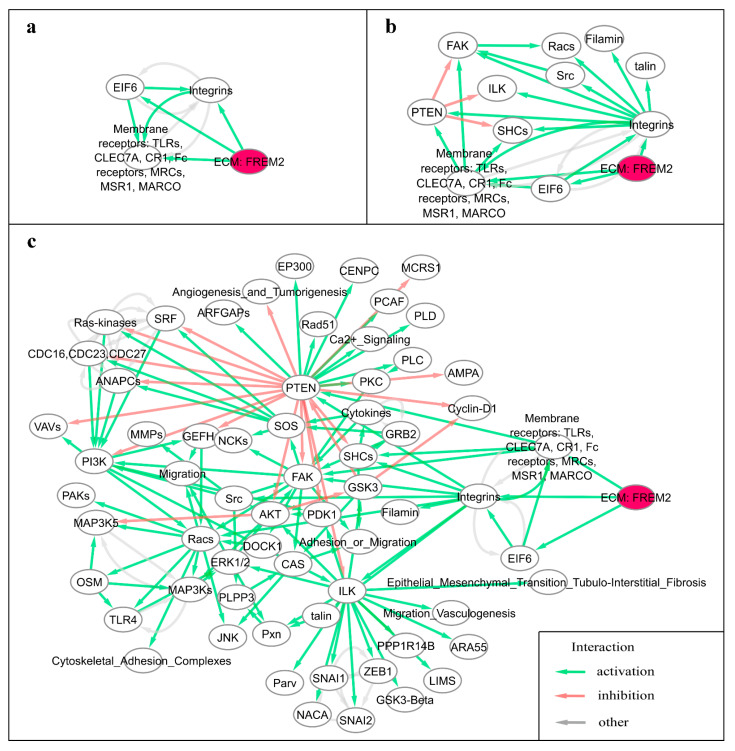
Algorithmically reconstructed variants of the *FREM2* pathway. (**a**) Variant 1 was created as a graph of first-order interactions with the *FREM2* gene product. (**b**) Variant 2 was created as a graph of first and second-order interactions with *FREM2*. (**c**) Variant 3 was created as a graph of first, second, and third-order interactions with *FREM2*.

We used the interactome model built to algorithmically identify interactions with *FREM2* protein. *FREM2* is included in the “extracellular matrix” node from five pathways considered: “Akt_Signaling_Pathway”, “ERK_Signaling_Pathway”, “ILK_Signaling_Pathway”, “MAPK_Signaling_Pathway”, and “PTEN_Pathway”. There were several downstream interactions with *FREM2*, but no upstream interactions were cataloged (Figure 3).

We reconstructed three variants of the *FREM2* pathway including sequentially interacting nodes for up to the third level of interactions starting from *FREM2*. As such, the first variant aggregated first-order interactions and included 4 nodes, 10 edges, and 53 gene products (Figure 3a). The second variant accumulated first-order and second-order interactions and contained 12 nodes, 26 edges, and 69 genes (Figure 2 and Figure 3b). Finally, the third variant combined first, second, and third-order interactions, with a total of 66 nodes, 147 edges, and 208 genes (Figure 3c). Activator/repressor roles were algorithmically calculated for every gene product in every pathway according to [30].

Pathway activation levels (PAL) were calculated for all three pathway types, for all tumor samples in every dataset, and were defined PAL1, PAL2, and PAL3, correspondingly. PAL positively reflects activation of a molecular pathway, where the absolute value of PAL reflects the extent of a pathway up/down regulation, and sign (+/-) of a PAL shown overall upregulation or inhibition of a pathway, respectively [37].

We then measured if these metrics (PAL1-3 and *FREM2* expression level) were connected with specific glioma conditions. To this end, we used the ROC AUC value as the measure of biomarker quality. The area under the ROC curve (AUC) is frequently used for scoring molecular biomarkers in oncology [38,39,40,41]. It reflects biomarker robustness and depends on its sensitivity and specificity [42]. It varies between 0.5 and 1, AUC less 0.7 reflects no biomarker ability to discriminate patients by condition, and 0.7 to 0.8 threshold is considered acceptable in diagnostic test assessment, 0.8 to 0.9 is considered excellent, and more than 0.9 is considered outstanding [43,44,45]. Thus, scoring *t*-test, *p*-value, and ROC AUC can answer two different questions: whether a metric under consideration is differentially regulated, and whether it can serve as a good biomarker.

### 3.5. FREM2 Gene and Pathways as LGG/GBM Grade Biomarkers

We investigated how the expression level of the *FREM2* gene or activation of *FREM2* pathways was connected with the LGG or GBM status of a tumor in the available datasets. For PAL values of all three variants of the *FREM2* pathway (*t*-test *p* < 1.08 × 10^−10^), and for *FREM2* expression levels (*p* < 4.46 × 10^−4^), we detected significant differences between LGG and GBM for all the relevant datasets tested (Table 1, Supplementary Figure S1).

However, in most datasets, *FREM2* expression returned AUC less than 0.7, and thus couldn’t be identified as the high-quality biomarker (Table 1). In contrast, all the FREM pathway variants demonstrated high AUC values (0.72–0.87) for discriminating LGG and GBM samples (Table 1). Interestingly, these *FREM2* pathway results were comparable with the performance of *IDH* mutation status as the biomarker, and significantly outperformed *MGMT* promoter methylation status as the biomarker (Table 1).

Thus, we conclude that all three variants of *FREM2* pathway activation are robust biomarkers for LGG and GBM discrimination, that have comparable performance with *IDH* mutation status, and significantly outperform *MGMT* methylation and *FREM2* expression as the biomarkers.

### 3.6. Performance of FREM2 Expression and Pathway Activation as OS and PFS Biomarker

**Figure 4:**
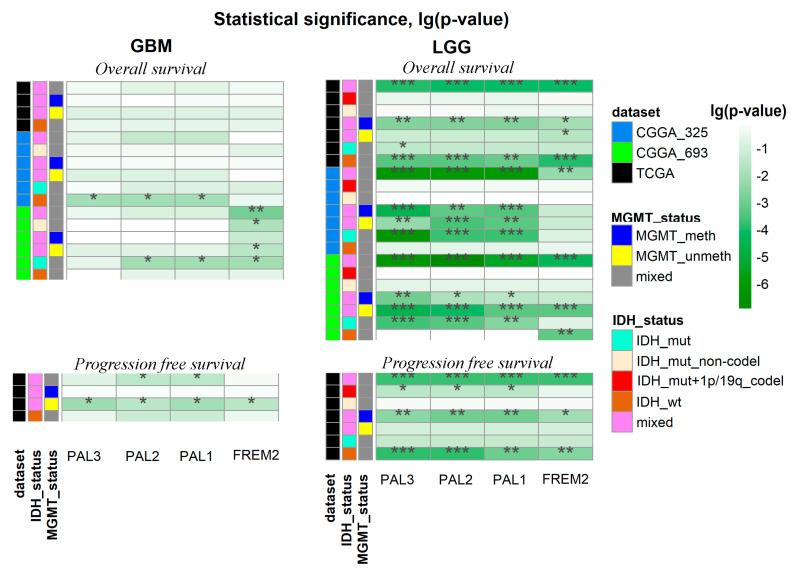
Performance of *FREM2*-based survival biomarkers in glioma datasets. Q-values of log-rank test of using *FREM2* expression (*FREM2*), PAL1, PAL2, and PAL3 were investigated as survival predictors in 48 comparisons. Each survival predictor was assessed relatively by its median (high level—the predictor was higher than its median and low level—the predictor was lower or equal to its median). * stands for q < 0.05, ** for q < 0.01, *** for q < 0.001. Color markers indicate dataset under analysis, *IDH* mutation, and *MGMT* promoter methylation statuses. “Mixed” stands for groups including samples with both variants of *IDH* or *MGMT* statuses. Status “IDH_mut” takes into account only IDH mutation existence, but not 1p/19q codeletion status.

We then investigated the performance of *FREM2* expression and pathway activation levels as the survival (OS and PFS) biomarker. To this end, we performed Kaplan-Meier analysis and calculated *p*-values of the log-rank test separately for GBMs and LGGs for all relevant datasets (Figure 4, Supplementary File 4).

Where possible, we also analyzed tumor molecular subgroups classified according to either *IDH* mutation or *MGMT* methylation status, the minimal size of a subgroup was ten samples. Smaller subsets were not considered for statistical significance reasons. The groups for Kaplan-Meier analysis were formed relatively median of *FREM2* variable (*FREM2* expression or *FREM2* pathway activation levels). Thus, we obtained groups with high-level samples (*FREM2* variable was higher than its median) and low-level samples (*FREM2* variable was lower or equal to its median) in each dataset or subgroup.

Thus, we performed a total of 48 comparisons for each possible biomarker under investigation (*FREM2* expression, PAL1-3) on different sets of glioma samples (Figure 4). In all the cases where statistically significant associations were found, the high *FREM2* pathway activation levels, or *FREM2* gene expression were linked with poor survival prognosis. We performed an FDR correction of *p*-values from a log-rank test because four potential biomarkers were tested to select the best of them. We found more statistically significant associations with the *FREM2* expression/pathway activation in the LGG datasets compared to the GBMs (Figure 4). Overall, *FREM2* expression was significantly associated (q-value < 0.05) with the survival characteristic in 33% of the comparisons with average log (q-value) −1.18. In turn, PAL1-3 were effective in 39.6%, 39.6%, and 37.5% of the cases, with average lg(q-value) −1.43, −1.51, and −1.47, respectively.

Thus, we concluded that the PAL2 was the best functional metric interrogated that resulted in the biggest number of statistically significant outputs (19 out of 48; 39.6%) and at the same time had the lowest average q-value for the statistical tests (Figure 4). For further investigations, we used PAL2 as it showed the best performance in previous tests.

**Figure 5:**
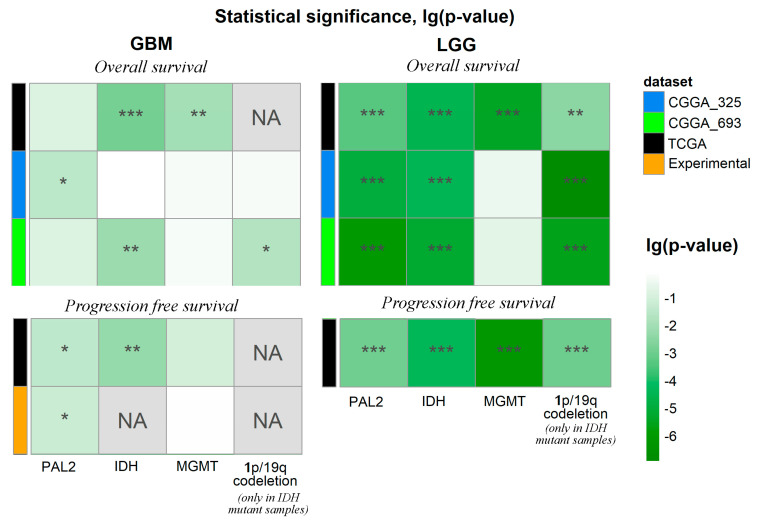
Performance of *FREM2* pathway PAL2 survival biomarker in glioma datasets in comparison with *IDH* mutation, *MGMT* methylation, and 1p/19q statuses. 1p/19q statuses were assessed only for *IDH* mutant tumors. *p*-values of log-rank test of using FREM PAL2, *IDH* mutation, and *MGMT* methylation statuses were investigated as survival predictors in 9 comparisons. * stands for *p* < 0.05, ** for *p* < 0.01, *** for *p* < 0.001. Color markers indicate the dataset under analysis. *IDH* mutation status was used for the full cohort, including samples with non-codeletion and 1p/19q-codeletion statuses.

We then compared the performance of *FREM2* pathway PAL2 with other well-known survival predictors: *MGMT* methylation and *IDH* mutation status (Figure 5).

To this end, these three predictors were tested in nine comparisons: in the literature glioma expression datasets (GBM and LGG from TCGA, CGGA_325, and CGGA_693 datasets—for OS and PFS), and one experimental GBM dataset for PFS. We used *p*-value without FDR correction here, because there is a descriptive comparison that presents the application area of three robust biomarkers on primary data.

In LGG samples, *FREM2* PAL2 and *IDH* mutation showed very good comparable performance for both OS and PFS analysis (Figure 5). In contrast, *MGMT* methylation status was a poor predictor in all LGG comparisons.

In GBM, neither of the biomarkers tested was effective in all three literature datasets for predicting the OS (Figure 5). However, for the PFS we were able to measure performance in only one literature dataset for *MGMT* and *IDH*, and in two datasets: experimental and literature—for PAL2. We found that *MGMT* methylation status was a poor predictor, but both *IDH* and *FREM2* PAL2 was effective as the GBM PFS biomarkers, and this was confirmed for *FREM2* PAL2 in an independent experimental validation study (Figure 5).

We then investigated if PAL2 is informative as an independent biomarker or if it simply reflects the *IDH* mutation status. To this end, we correlated *FREM2* PAL2 with *IDH* mutation status in all available datasets. We detected statistically significant negative correlations for both LGG (−0.28; −0.36) and GBM (−0.09; −0.52), with a mean correlation of −0.31 (Supplementary File 5). We, therefore, conclude that although the correlations were statistically significant and supported common trends traced by the PAL2 and *IDH* biomarkers, still their extent was relatively low. Thus, our findings support PAL2 as the independent biomarker.

We also detected good effectiveness of PAL2 (*p* < 0.05) for PFS prognosis inside four LGG subgroups: with wt*IDH,* mutant *IDH*, mutant *IDH* with 1p/19q codeletion (*p* < 0.05) and methylated *MGMT*, and in GBM subgroups with wt*IDH* and with unmethylated *MGMT* promoter. This further confirms the independent utility of the PAL2 biomarker in gliomas.

In total, we identified five glioma conditions where *FREM2* pathway PAL2 effectiveness was confirmed in two or more available datasets as OS or PFS predictor (Table 2): (*i*) for OS in LGG, (*ii*) for OS in LGG with methylated *MGMT*, (*iii*) for OS in LGG with unmethylated *MGMT*, (*iv*) for OS in LGG with *IDH* mutation; (*v*) for PFS in GBM. We used *p*-value without FDR correction in Table 2 because there is a descriptive presentation of how one biomarker works on primary data, these cases are complementary and define the application area of the new biomarker.

**Figure 6:**
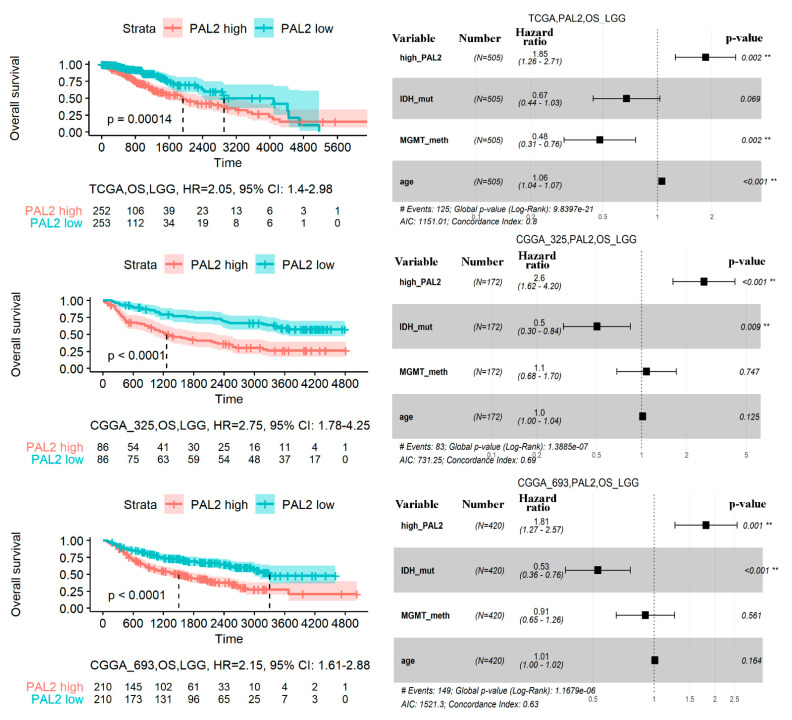
Kaplan-Meyer, univariate and multivariate Cox analyses of OS in LGG for three literature datasets (TCGA, CGGA_325, and CGGA_693). The samples were grouped by PAL2 level relatively to PAL2 median value (“high” PAL2: PAL2 > median(PAL2); “low” PAL2: PAL2 < median(PAL2)). Time is given in days in all graphs. ** stands for *p* < 0.01.

For these glioma subtypes, we calculated *hazard ratio* values from univariate Cox models and built Kaplan-Meyer plots (Supplementary Figure S2). For OS in LGG, the effectiveness of PAL2 was confirmed in the multivariate Cox model for all three available datasets. The model included PAL2, age, *IDH* mutation status, and *MGMT* methylation status (Figure 6).

**Figure 7:**
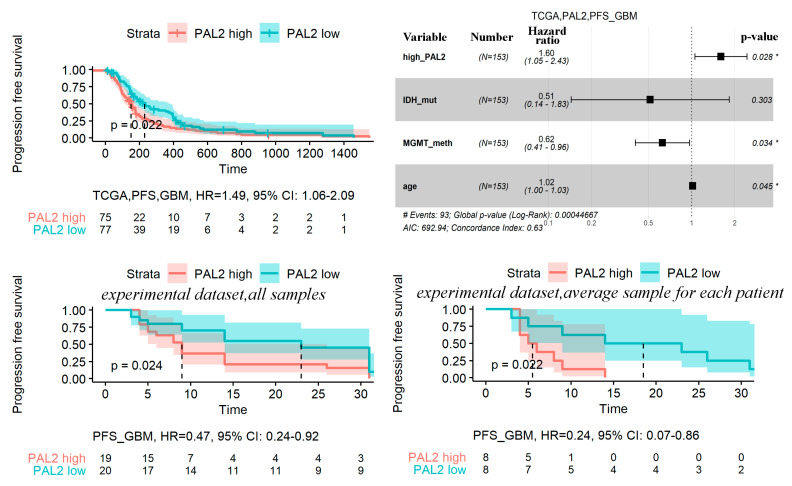
Kaplan-Meyer, univariate and multivariate Cox analyses of progression-free survival in GBM for TCGA. Kaplan-Meyer and univariate Cox analyses of progression-free survival in GBM for experimental dataset (for all samples and for averaged (by patient) samples). The samples were grouped by PAL2 level relative to the median value of PAL2 (“high” PAL2: PAL2 > median (PAL2); “low” PAL2: PAL2 < median (PAL2)). Time is given in days for the TCGA dataset, and in months for the experimental dataset. * stands for *p* < 0.05.

The link between PAL2 and PFS in GBM was validated on the experimental dataset. The experimental dataset included 39 GBM samples from 16 patients, where for 12/16 of the patients’ several tumor samples were profiled by RNAseq. Duplicated tumor samples were obtained from different regions from the same tumor. For each patient, PFS and *IDH* mutation status were measured, but only for 11 patients, *MGMT* methylation status was known (Supplementary File 1). We, therefore, tested the performance of *FREM2* pathway PAL2 in two variants: (*i*) using the expression profile of each sample separately, and (*ii*) using averaged expression profile for every patient. In both types of the analysis, high PAL2 values were associated with the poor prognosis on PFS in GBM patients (*p* < 0.05; Figure 7).

**Figure 8:**
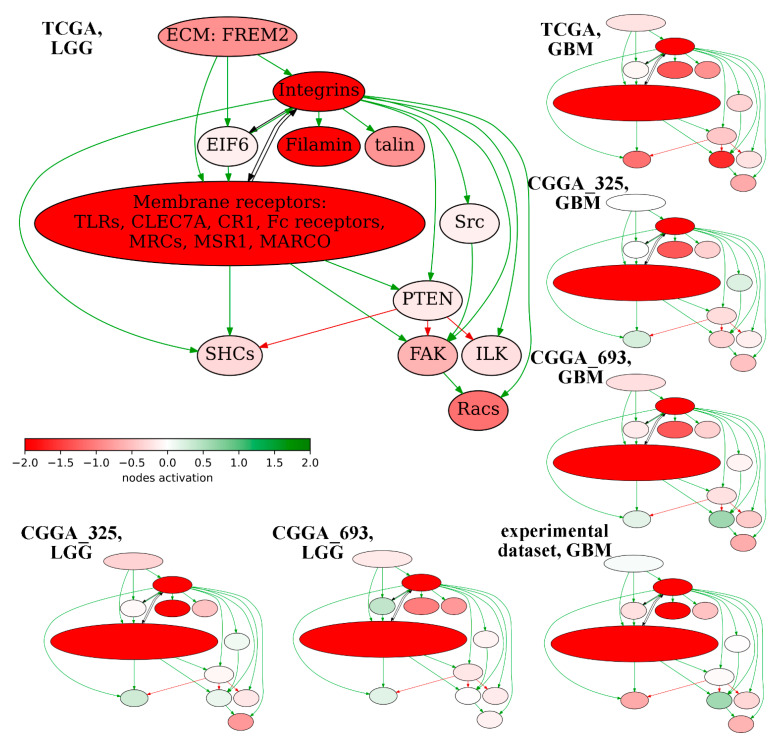
*FREM2* pathway activation profile built for seven available glioma expression datasets. Node activation is a logarithmic ratio of expression in the averaged sample with low PAL2 (~good prognosis) to expression in the averaged sample with high PAL2 (~bad prognosis). Green arrows denote activation, red—inhibition, black –other interactions.

We then analyzed expression profiles of the reconstructed *FREM2* pathway components to assess their impacts on the pathway activation levels. The *FREM2* pathway activation profiles were built using the Oncobox platform [41] for each available dataset. For visualization, gene expression levels in an averaged sample with low PAL2 (good prognosis) were normalized on expression levels in the averaged sample with high PAL2 (poor prognosis), Figure 8. Overall, we observed very similar activation profiles for different datasets interrogated, with little variation for LGG and GBM samples.

### 3.7. FREM2 Gene and Pathway as Molecular Subtype Biomarkers

We then investigated how the expression level of the *FREM2* gene or activation of the *FREM2* pathway were connected with molecular subtypes of GBM (mesenchymal, classical, proneural). For *FREM2* pathway PAL2 values (*t*-test *p* < 1.23 × 10^−5^, AUC > 0.71) we detected a significant difference between mesenchymal and other subtypes for all relevant datasets tested (Table 3, Supplementary Figure S3). However, *FREM2* pathway activation level was not associated with overall or progression-free survival within each molecular subtype in all three literature datasets investigated.

There was also a tendency of differential *FREM2* gene expression in classical subtype vs others (*t*-test *p* < 0.05), but AUC was less than 0.7 for most of the comparisons. Thus, *FREM2* gene expression cannot serve robust biomarker for GBM molecular subtypes (Table 3, Supplementary Figure S3).

**Figure 9:**
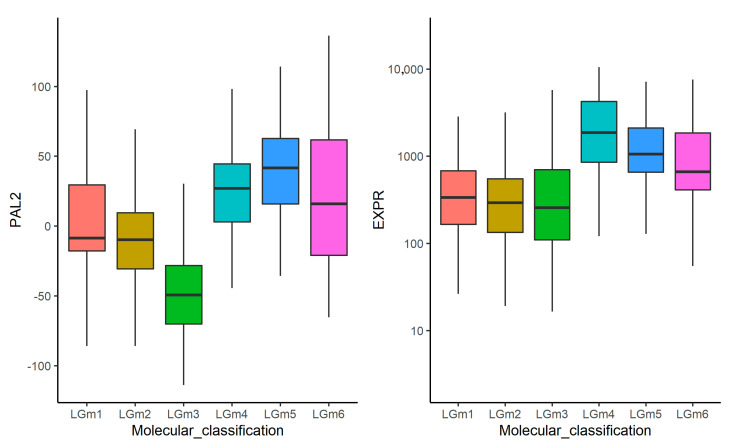
Boxplots for *FREM2* gene expression and pathway activation level (PAL2) in comparison between glioma molecular subtypes LGm1-6 from [19]. Only the TCGA dataset was used.

Likewise, we explored how *FREM2* gene expression and pathway activation can discriminate glioma molecular subtypes LGm1-6 which are described in Ceccarelli M. et al. [19] and strongly associated with the DNA methylation-based classification of gliomas [46]. Only the TCGA dataset was used for these analyses because other datasets contained no relevant tumor methylation data. We found that *FREM2* gene expression and PAL2 were differential in these subtypes (Kruskal-Wallis test *p* < 1.3 × 10^−39^ and *p* < 2.1 × 10^−59^, respectively (Figure 9)). Higher PAL2 was associated with lower OS in LGm6 (*p* = 0.032), and lower PFS in LGm3 and LGm4 (*p* < 0.01).

## 4. Discussion

We report here a new reconstructed *FREM2* molecular pathway, which activation is strongly associated with unfavorable prognosis in glioma patients. We have tested three algorithmically built agnostic variants of this pathway and selected the best version in 34 independent comparisons. This survival predictor capacity of *FREM2* pathway activation was robust and comparable with *IDH* mutation status, but significantly superior to *MGMT* methylation status. Moreover, *FREM2* pathway activation level (PAL) could effectively predict survival within the subgroups with different *IDH* mutation statuses: OS in *IDH* mutant LGG, and PFS in *IDH* mutant GBM, in LGG with wt*IDH,* and LGG with mutant *IDH*. Furthermore, activation levels of the *FREM2* pathway were significantly higher in GBM than in LGG samples.

Expression of *SPRY1* gene, another potential glioma biomarker that showed comparable characteristics to *FREM2* in the previous study [15], was also associated with survival and showed a similar pattern to *FREM2* expression (Supplementary File 6). However, the *SPRY1* pathway hasn’t been reconstructed and investigated yet, and we plan that this will be a matter of our further studies.

As reported previously, *FREM2* is associated with mesenchymal differentiation in gliosarcoma because it was strongly overexpressed in mesenchymal compared to glial tumor areas. [47]. In addition, increased *FREM2* gene expression was demonstrated in gliomas compared to the normal glia, and in GBM compared in LGG [15,16,48]. We found no other reports on *FREM2* implication in cancers.

However, other genes of the same gene family as *FREM2*, namely *FRAS1* and *FREM1,* were recently reported as cancer-related genes. In particular, knockout of *FRAS1* inhibits proliferation of gastric cancer cells through caspase activity increment and cell cycle arrest both in vitro and in vivo [49]. Conversely, increased expression of *FREM1* in breast cancer is associated with a favorable prognosis and high-level immune infiltration status [50].

Among the reconstructed *FREM2* pathway members we detected strongly upregulated expression of integrin family members, filamin, and several types of membrane receptors associated with the poor survival prognosis. Thus, we propose that these components of the *FREM2* pathway are important actors of glioma pathogenesis and could be regarded as the possible new targets for next-generation molecular therapeutics.

Indeed, integrins were previously mentioned as the potential targets of GBM therapy because of their major role in tumor invasion and strongly differential expression [51,52,53]. Unfortunately, small molecule integrin antagonists did not meet high expectations in GBM therapy [51]. Instead, further investigation of the *FREM2* pathway can help to find and validate additional molecular targets that could be affected in a combinational therapy of malignant gliomas.

Next-generation sequencing technologies allowed us to get a better insight into the molecular biology of GBM, but also to identify new disease-specific changes and molecules. However, despite extensive research, the life expectancy of GBM patients has not significantly improved in decades. Primary GBMs are ranked first among cancer types in years of life lost—on average 20.1 years compared to 11.8 years for lung cancer and 6.8 years for prostate cancer [54]. Currently, there are only two predictive biomarkers, in particular, *MGMT* promoter methylation and 1p/19q codeletion. In older patients presenting with wt*IDH* glioblastoma, the presence of *MGMT* promoter methylation predicts a positive response to therapy and longer survival [55,56,57]. Chromosome 1p/19q codeletion is suggested as a beneficial biomarker in elderly patients when they receive combined radiation and chemotherapy with procarbazine CCNU vincristine (PCV). The improvement in overall survival was also proved with two phase III clinical trials that showed a 2-fold increase in median survival in patients with 1p/19q codeletion [58,59]. Prediction of survival and progression of glioblastoma patients can be done by investigating changes in structural magnetic resonance imaging (MRI) [60] and implementing various machine learning models [61]. However, even with the growing number of prognostic models, the clinical implementation of models for predicting the prognosis of glioblastoma patients remains difficult [62]. Thus, the need for the identification of reliable biomarkers for diagnostic, prognostic, and therapeutic purposes remains unchanged [63].

## 5. Conclusions

Because of its robustness and survival predictor capacity, the *FREM2* pathway can be used for adjustment of treatment schedule that will result in a better quality of life of patients and optimization of costs. Owing to the large sample size used in this study, we believe that the *FREM2* pathway shows the potential to be easily implemented in routine practice after validation in clinical settings.

This biomarker holds the potential to be used as a predictive biomarker of high potential benefit for the patients. On that account, because it was found robust, comparable to *IDH* mutation status, and superior to *MGMT* methylation status in most of the comparisons, we propose *FREM2* pathway activation as a novel robust predictor of unfavorable prognosis of glioblastoma patients.

## Figures and Tables

**Table 1:** Performance of *FREM2* expression and *FREM2* pathway activation for discrimination of LGG and GBM tumor samples.

	*t*-Test *p*-Value					
	PAL3	PAL2	PAL1	*FREM2*	*IDH* Mutation	*MGMT* Methylation
TCGA	4.35 × 10^−30^	1.92 × 10^−47^	4.23 × 10^−49^	4.68 × 10^−7^	7.75 × 10^−91^	2.51 × 10^−14^
CGGA_325	1.08 × 10^−10^	1.63 × 10^−13^	7.74 × 10^−13^	4.46 × 10^−4^	3.66 × 10^−16^	3.81 × 10^−1^
CGGA_693	4.99 × 10^−25^	1.49 × 10^−28^	1.77 × 10^−27^	4.57 × 10^−5^	1.86 × 10^−49^	8.36 × 10^−2^
	**AUC**					
	**PAL3**	**PAL2**	**PAL1**	***FREM2***	***IDH* Mutation**	***MGMT* Methylation**
TCGA	0.80	0.86	0.87	0.76	0.83	0.7
CGGA_325	0.72	0.74	0.73	0.66	0.72	0.53
CGGA_693	0.74	0.75	0.75	0.66	0.78	0.54

**Table 2:** Effectiveness of *FREM2* pathway PAL2 as survival prognosis factor. “+” means statistically significant difference in comparison by the *p*-value of the log-rank test, “−” means non-significant comparisons. Positive results that coincide with the available datasets are shaded.

Cancer Type and Functional Characteristic Assessed	Interrogated Dataset			
	TCGA (*n* = 153 GBM, 505 LGG)	CGGA_325 (*n* = 137 GBM, 172 LGG)	CGGA_693 (*n* = 237 GBM, 420 LGG)	Experimental (*n* = 39 GBM)
OS, GBM	−	+	−	N/A
OS, GBM, *MGMT* methylated	−	−	−	N/A
OS, GBM, *MGMT*_unmethylated	−	−	−	N/A
OS, GBM, *IDH* mutated	N/A	−	+	N/A
OS, LGG, *IDH* mutated without 1p/19q codeletion	N/A	−	−	N/A
OS, GBM, *IDH* wild-type	−	+	−	N/A
**OS, LGG**	**+**	**+**	**+**	**N/A**
**OS, LGG, *MGMT* methylated**	**+**	**+**	**+**	**N/A**
**OS, LGG, *MGMT*_unmethylated**	**−**	**+**	**+**	**N/A**
**OS, LGG, *IDH* mutated**	**+**	**+**	**+**	**N/A**
OS, LGG, *IDH* wild-type	+	−	−	N/A
OS, LGG, *IDH* mutated+1p/19q codeletion	−	−	−	N/A
OS, LGG, *IDH* mutated without 1p/19q codeletion	−	−	−	N/A
**PFS, GBM**	**+**	**N/A**	**N/A**	**+**
PFS, GBM, *MGMT* methylated	−	N/A	N/A	N/A
PFS, GBM, *MGMT*_unmethylated	+	N/A	N/A	N/A
PFS, GBM, *IDH* mutated	N/A	N/A	N/A	N/A
PFS, GBM, *IDH* wild-type	+	N/A	N/A	N/A
PFS, LGG, *IDH* mutated+1p/19q codeletion	+	N/A	N/A	N/A
PFS, LGG, *IDH* mutated without 1p/19q codeletion	−	N/A	N/A	N/A
PFS, LGG	+	N/A	N/A	N/A
PFS, LGG, *MGMT* methylated	+	N/A	N/A	N/A
PFS, LGG, *MGMT* unmethylated	−	N/A	N/A	N/A
PFS, LGG, *IDH* mutated	+	N/A	N/A	N/A
PFS, LGG, *IDH* wild-type	+	N/A	N/A	N/A

**Table 3:** Performance of *FREM2* expression and *FREM2* pathway activation for discrimination of GBM molecular subtypes.

	*t*-Test *p*-Value					
	PAL2			*FREM2* Expression		
	Mesenchymal vs. Proneural	Proneural vs. Classical	Mesenchymal vs. Classical	Mesenchymal vs. Proneural	Proneural vs. Classical	Mesenchymal vs. Classical
TCGA	3.59 × 10^−13^	6.11 × 10^−2^	4.28 × 10^−10^	2.39 × 10^−1^	7.69 × 10^−3^	1.60 × 10^−4^
CGGA_325	1.67 × 10^−4^	9.48 × 10^−1^	4.18 × 10^−10^	6.45 × 10^−1^	2.25 × 10^−2^	2.61 × 10^−1^
CGGA_693	1.23 × 10^−5^	8.40 × 10^−1^	1.23 × 10^−5^	8.45 × 10^−1^	2.17 × 10^−2^	8.47 × 10^−2^
	**AUC**					
	**PAL2**			***FREM2* Expression**		
	**Mesenchymal vs. Proneural**	**Proneural vs. Classical**	**Mesenchymal vs. Classical**	**Mesenchymal vs. Proneural**	**Proneural vs. Classical**	**Mesenchymal vs. Classical**
TCGA	0.90	0.61	0.85	0.56	0.66	0.73
CGGA_325	0.86	0.52	0.87	0.58	0.67	0.75
CGGA_693	0.71	0.50	0.72	0.56	0.73	0.69

## Data Availability

The expression data of 39 biopsy specimens were deposited in the NCBI Sequencing Read Archive (SRA) repository with ID SRP227324.

## Supplementary Materials

The following are available online at https://www.mdpi.com/article/10.3390/cancers13164117/s1, Figure S1. Boxplots for each type of *FREM2* expression metric (gene expression, PAL1, PAL2, PAL3) in comparison between LGG and GBM tumor samples. Every panel contains a t-test *p*-value for the corresponding dataset. Figure S2. Kaplan-Meyer test for selected subgroups of gliomas: GBM or LGG with *IDH* mutation, with methylated *MGMT*, with unmethylated *MGMT*. The samples were grouped by PAL2 level relatively to PAL2 median (“high” PAL2: PAL2 > median (PAL2); “low” PAL2: PAL2 < median (PAL2)). Only comparisons with *p*-value < 0.05 in two or three parallel datasets were plotted. A full list of comparisons performed is given in Table 2. Figure S3. Boxplots for *FREM2* gene expression and pathway activation level (PAL2) in comparison between glioblastoma molecular subtypes: classical, mesenchymal, proneural in TCGA, CGGA_325, CGGA_693 datasets. Supplementary File 1. Clinical information for experimental GBM dataset: patient ID, diagnosis, *IDH* mutation status, *MGMT* promoter methylation status, type of therapy, time to progression (months). Supplementary File 2. Statistics of RNAseq reads mapping for the experimental dataset. Supplementary File 3. *FREM2* pathway PAL2 values for the samples from the literature and experimental glioma expression datasets. Supplementary File 4 Histological composition of LGG subgroups from 48 comparisons in Figure 4 and Figure 5, and Table 2. Supplementary File 5. Correlation statistics for PAL2 with *IDH* mutation, *IDH* mutation+ 1p/19q co-deletion or with *MGMT* promoter methylation statuses. Supplementary File 6. Performance of *FREM2* and *SPRY1* expression as survival biomarkers in glioma datasets. Each survival predictor was assessed relatively by its median (high level—the predictor was higher than its median and low level—the predictor was lower or equal to its median).
