# Supplementary figures and images for: Algorithmically Deduced FREM2 Molecular Pathway Is a Potent Grade and Survival Biomarker of Human Gliomas
Source: Cancers (Basel). 2021 Aug 16;13(16):4117. doi: 10.3390/cancers13164117 (PMC8394245; doi:10.3390/cancers13164117)

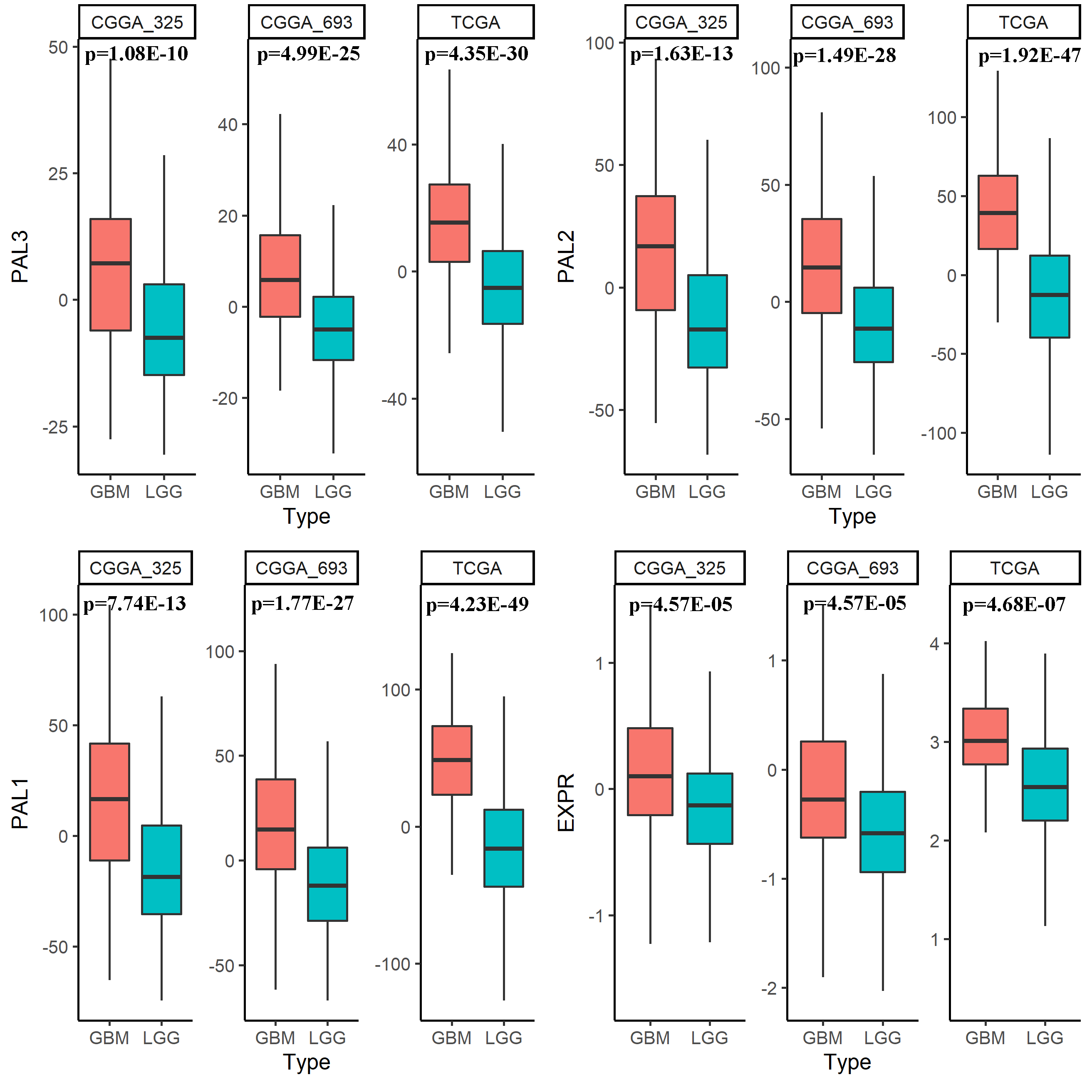

Supplement: Supplementary file 1 [file cancers-13-04117-s001.zip › Supplementary_Figure_S1.tif]

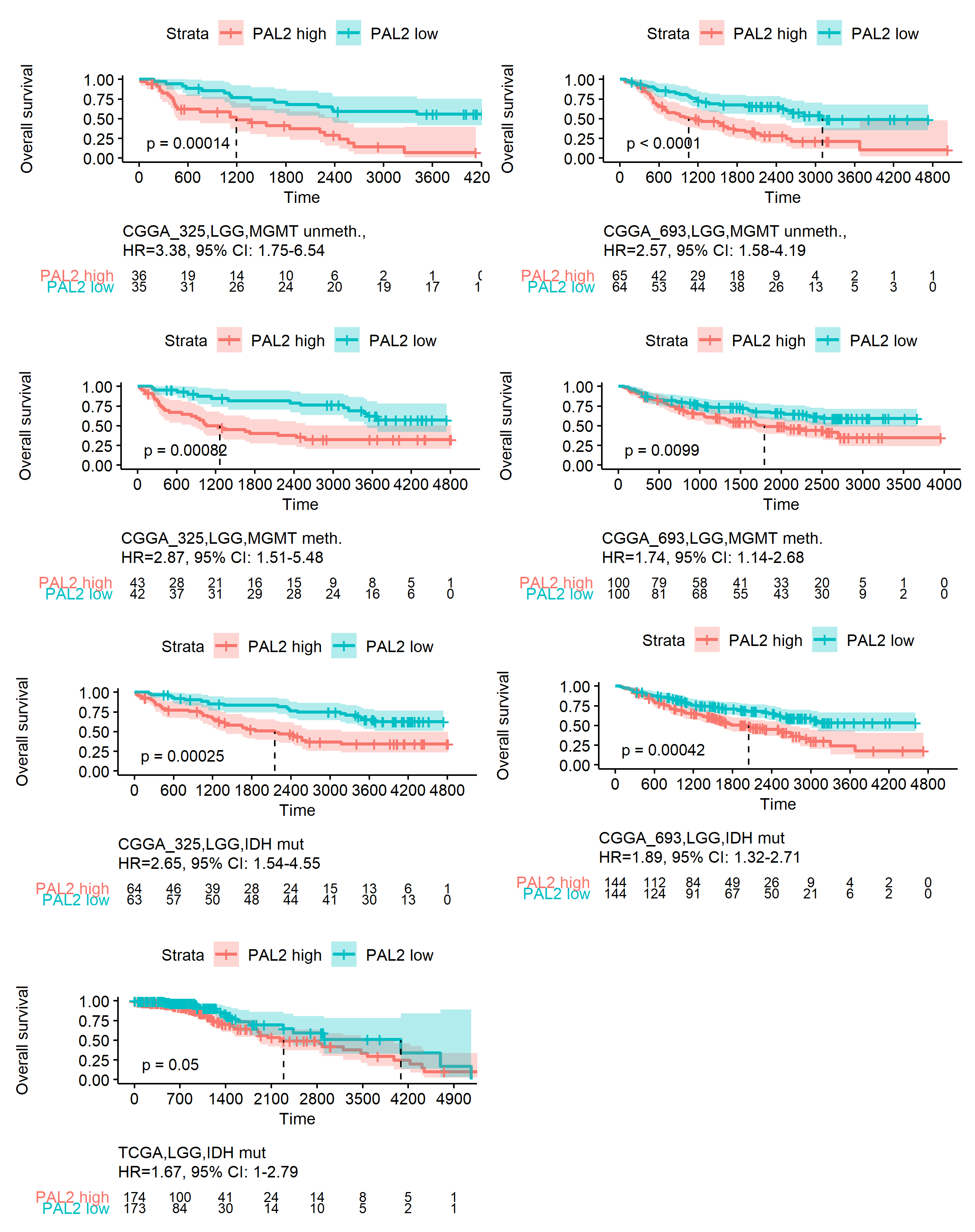

Supplement: Supplementary file 1 [file cancers-13-04117-s001.zip › Supplementary_Figure_S2.tif]
